# Induced Biological Response in Contact with Ag-and Cu-Doped Carbon Coatings for Potential Orthopedic Applications

**DOI:** 10.3390/ma14081861

**Published:** 2021-04-09

**Authors:** Krzysztof Jastrzębski, Jerzy Białecki, Aleksandra Jastrzębska, Anna Kaczmarek, Marcin Para, Piotr Niedzielski, Dorota Bociaga

**Affiliations:** 1 Institute of Materials Science and Engineering, Lodz University of Technology, 1/15 Stefanowskiego St., 90-924 Lodz, Poland; jastrzebska.aleksandra1@gmail.com (A.J.); piotr.niedzielski@p.lodz.pl (P.N.); dorota.bociaga@p.lodz.pl (D.B.); 2 Ortopaedic Clinic of Centre of Postgraduate Medical Education in Otwock, Konarskiego 13, 05-400 Otwock, Poland; jerzybialecki@gmail.com (J.B.); marcinpara@gmail.com (M.P.); 3 Lukasiewicz Research Network-Textile Research Institute, Brzezinska 5/15, 92-103 Lodz, Poland; ania.m.olejnik@gmail.com

**Keywords:** Saos-2, EA.hy926, DLC, bactericidal effect, silver admixture, copper admixture

## Abstract

Silver and copper as additives of various biomaterials have been reported as the potential solutions for biomedicine applications, mostly because of inducing bactericidal effects. The application of those admixtures in diamond-like carbon (DLC) coatings may be desirable for orthopedic implants. In the present manuscript, the biological effect of coatings with up to about 7 at.% and 14 at.% of, respectively, Cu and Ag is compared. The morphology, chemical structure, and composition of films deposited on AISI 316LVM and Ti6Al7Nb is characterized. The live/dead analysis conducted with *Escherichia coli* shows a higher bactericidal potential of silver than copper. Although the Cu-doped coatings can positively affect the proliferation of Saos-2 and EA.hy926 cell lines, the results of XTT test are on the verge of 70% of viability. Biological effect of silver on EA.hy926 cell lines is negative but that admixture ensures high proliferation of osteoblasts in contact with coatings deposited on titanium alloy (over 20% better than for substrate material). In that case, the viability is reaching about 85% for Ag-doped coatings on AISI 316LVM and 75% on Ti6Al7Nb. The results indicate that for the sake of bactericidal coatings that may promote osteointegration, the candidates are DLC with silver content no higher than 10 at.%.

## 1. Introduction

About 1.5 million alloplastic procedures are made in Europe every year; similar is also the number of implants used in traumatology [1]. What is more, the market of hip and knee replacement is worth approximately 25% of the global orthopedics market [2]. The increasing number of surgical procedures performed in orthopedics and traumatology, in particular arthroplasty, and the high number of revision operations in that field, have resulted as a consequence of peri-implant infections. In that manner, post operational complications related to infections are the third most common cause of failures in endoplastic surgery and the second most common cause of revision surgeries [3].

Surgical site infections and inflammatory complications of osteosynthesis affect from 1 to 14% of all treated patients. What is more, the forecasts of total cost of treating periprosthetic inflammation in Markov’s analysis in the US is approximately $390,000, while the costs of one-time hospitalization of a patient with periprosthetic inflammation exceeds $31,000 in that country [4,5]. These numbers significantly increase the cost of treatment and, as a result, cause a very heavy financial burden on health systems. The obvious social problems resulting from these issues are dissatisfaction of operated-upon patients and possible future disabilities. Seeking opportunities to reduce the risk of inflammatory-related complications, activities need to focus on the improvement in peri-operative and surgical prevention of these problems. Among others, research is conducted in order to protect implants against microbial colonization, and microbial colonization by means of various implant coatings.

Romano et al. [6] proposed the division of implants used in orthopedics into active antibacterial—these are antibacterial through releasing bactericidal substances; passive, in which the material itself—its structure—inhibits bacterial adhesion; and finally into intraoperative specimens, that protect implants during surgery.

The first group primarily includes implants coated with metal ions such as silver, copper, or zinc or bactericidal compounds such as tincture of iodine. The second group consists of passive materials that reduce bacterial adhesion to the implant due to structure modifications like titanium dioxide or polymer coatings. Among specimens used for coating implants during surgery are gels such as defensive antibacterial coating or bone grafts soaked with antibiotics [6,7].

Implants can be coated with body fluids with serum proteins such as fibrinogen, fibronectin, albumin, vitronectin, and others [8,9]. Those compounds compete with microorganisms for adhesion to the implant surface. This phenomenon was called by Gristina et al. as “the race for the surface” [10,11]. If this competition is won by microbial cells, they colonize the implant, creating on its surface a biofilm. Such structure is highly resistant to antibiotics and, by production and accumulation of toxin or inhibitory factors, reduces the activity of host cells impairing osseointegration [12,13].

One of the methods often used to improve antibacterial properties of the implants surfaces is also the use of carbon layers. The commonly used name for that family of coatings is DLC (diamond-like carbon) that refers to a wide variety of materials made of amorphous carbon. The basic factors influencing their properties are the ratio of the number of sp2 and sp3 bonds and the amount of hydrogen present in the structure. Their biological potential may be improved by doping with elements known from their anti-inflammatory or antibacterial properties such as silver, copper silicone [14], fluorine [15], and even germanium [16]. Among currently developed methods of manufacturing of those layers in use are: magnetron sputtering [17,18], electron cyclotron resonance-discharge cleaning (ECR-DC) [19], hybrid radio frequency/magnetron sputtering plasma-assisted chemical vapor deposition (RF/MS PACVD) [20], and electrochemical methods [21]. Thin carbon films doped with Ag, Cu, Si, etc., in addition to affecting the biological properties, also exhibit interesting functional and mechanical properties. The main advantages of this type of layers are the improvement of tribological properties, hardness, and adhesion of coatings to the substrate [22,23].

When silver or copper are used as admixtures of carbon coatings, the exerted bacteriostatic or bactericidal effect is primarily related to the effect of these metals or their ions on the cells of microorganisms and not the change of surface morphology. Usually, silver content in the coating of up to 10% are tested, although in some cases it exceeds 25% [24]. Ag-DLC coatings successfully limit the development of microorganisms responsible for diseases that are dangerous to humans, e.g., *Staphylococcus aureus*. Silver-containing carbon coatings reduce the amount of bacteria even within a few hours of contact. Baba et al. [24] indicated an antibacterial effect of over 60% reduced bacteria viability after 3 h of incubation. However, the presence of silver ions or silver nanoparticles at concentrations that cause a toxic effect in bacteria, may significantly reduce the viability of cells [25,26], e.g., mezynhymal human stem cells [27]. In case of copper, depending on the bacterial strain used in microbiological tests, the bactericidal effect may increase with an increasing amount of admixture (for *Bacillus subtilis*), or the opposite trend (*Escherichia coli*) can be noticeable [28]. Publications describing the properties of Cu-DLC coatings do not focus on the effects of this admixture on human tissues or even their animal models. The presence of copper, however, undoubtedly affects, for example, the skeletal system [29,30] and the bloodstream [31].

Introduction of any type of coating protecting from microbial adhesion and biofilm formation (such as those including silver and copper) can also affect the surrounding tissues. That may involve positive effects such as a stimulation of osteoblasts leading to improved osteointegration [32]. On the other hand, they may also exert unexpected complications. Release of many elements from the surface can potentially cause toxic effects, damaging primarily the bone marrow and kidneys. Furthermore, local effects can lead to osteolysis in the area of implantation and loosening of implants that leads to further negative consequences. Therefore, it seems necessary to verify the influence of Cu-DLC and Ag-DLC coatings on the proliferation and viability of human cells, like osteoblasts.

The main aim of this work was to compare the antibacterial effectiveness and biological safety of two kinds of DLC coatings: doped with silver and with copper. For this purpose, a microbiological assessment was conducted using *E. coli* model organism and in vitro evaluation with two cell lines, namely endothelial (EA.hy 926) and osteogenic (Saos-2) ones. The results of biological evaluation were correlated with, e.g., chemical and bond structure of the films manufactured by means of magnetron sputtering method.

## 2. Materials and Methods

### 2.1. Coatings Deposition

All tested coatings were deposited by means of magnetron sputtering method with use of deposition chamber (Prevac sp. z.o.o, Rogów, Poland) equipped with 3 sputtering guns. Graphite target was used as a carbon source and metal (Ag, Cu) target for introduction of admixture. The power used for sputtering of silver and copper was not higher than 10 W. The manufactured samples were denoted as X-DLCY, where X stands for additive element (Ag or Cu), while Y is the number encoding increasing concentration of the added element. The coatings doped with the same element, deposited on various substrate materials, and denoted with the sign Y, were deposited using congruous process parameters. The procedure of coatings manufacturing was described in details in other publication [33]. The adopted process parameters (including the sputtering power for the admixture in the range up to 0.5 W/cm^2^) allowed to obtain coatings of thickness up to 200 nm.

The materials currently used for the production of implants and medical instruments were selected as substrates, namely: AISI 316 LVM austenitic steel and Ti6Al7Nb titanium alloy. In each case, samples of 16 mm in diameter and height of 6 mm were mechanically ground and mirror-polished before the deposition. Additionally, processes of cleaning and degreasing of samples were performed ultrasonically in acetone.

### 2.2. Scanning Electron Microscopy (SEM)

Scanning Electron Microscopy (SEM) was used to visualize and evaluate the topography of all examined samples. SM-6610LV (JEOL, Peabody, MA, USA) equipped with X-Max 80 energy dispersive X-ray spectroscopy (EDS) analyzer (Oxford Instruments, Abingdon, UK) was used for conducting observations with secondary imaging mode (SEI—secondary electron image) acquiring topographic images. SEM observations of randomly selected samples were carried out under high vacuum with an electron beam acceleration voltage of 20 kV and 1000× magnification in random spots.

### 2.3. X-ray Photoelectron Spectroscopy (XPS)

The assessment of the surface chemistry of examined materials (chemical composition, characterization of bond types, and determination of sp^2^/(sp^2^ + sp^3^) ratio were performed by means of XPS method. The AXIS Ultra DLD (Kratos Analytical) system was used, equipped with an aluminum electrode (Kα = 1486.6 eV) working at 14.5 kV and 20 mA as a non-monochrome X-ray source. The data acquisition time was 151 s, energy step equaled to 100.0 meV, and waiting time was 250 s. Three randomly selected places on the examined surfaces were used for the analysis. Wide-scan spectra were performed in the range from 0 to 1200 eV. The molecular architecture of the tested materials was evaluated by examination of characteristic peaks of carbon and admixture elements with a resolution of 0.1 eV:

- C_1S_ peak corresponding to the energy of 284.6 eV (deconvolution was performed using Gauss–Lorentz curves corresponding to the peaks characteristic for sp^2^ C=C (284.5 eV) and sp^3^ C–C (285.3 eV) bonds, as well as C–O (286.1 eV) and C=O (288 eV) bonds,
- Cu_2p_ peak (922–970 eV) corresponding to the region characteristic (922–970 eV) for copper admixture,
- Ag_3d_ (922–970 eV) corresponding to the region characteristic for silver admixture.

### 2.4. Wettability and Surface Free Energy (SFE)

The sitting drop method was used for the assessment of coatings wettability, and the Owens–Wendt method was used to determine the surface free energy (SFE) of the tested materials. Surface energy was treated as the sum of two components: polar and dispersive ones, which can be calculated using measurements of contact angles for liquids with known parameters. The selected liquids were distilled water-polar liquid, and diiodomethane–non polar liquid.

The measurements of contact angles were performed with use of FM40 Easy Drop system with Drop Shape Analysis software (Krüss GmbH, Krüss GmbH, Germany), enabling the accuracy of 0.1°. Three samples of each type of modification were tested and, in each case, the measurements were conducted in triplicate.

### 2.5. Assessment of Microbial Colonization

LIVE/DEAD staining was used to assess surface colonization by the model organism *Escherichia coli*. Samples of all the examined coatings (3 from each type) after steam sterilization were incubated for 24 h at 37 °C in 200 mL of liquid culture medium. The 10-minutes long staining was performed with use of two fluorescent dyes: bis benzimidine (HOECHST 33258; Ex = 345 nm, Em = 487 nm) and propidium iodide (Ex = 538 nm, Em = 617 nm). Bis-benzamidine penetrates the cell membrane and then binds to double-stranded DNA in an area called a small groove and serves for the staining of live cells. Propidium iodide, due to its charge, does not pass the cell membrane of healthy microorganisms. However, it easily binds to single-stranded DNA or RNA of necrotic or apoptotic cells with damaged membranes. Bacterial cells were observed using a fluorescence microscope (Olympus GX71 equipped with a DP70 digital camera). On each of the three analyzed samples of the same type, 7 pictures were taken at random locations. The number of live (green-colored) and dead (red-colored) cells were counted using freeware software Image J.

Uncoated samples-C-were used as the control for assays of microbiological activity of Ag and Cu elements. In case of coatings deposited on Ti6Al7Nb, the additional control-AISI 316 LVM was also used in order to compare these two metallic substrates.

### 2.6. In Vitro Evaluation with Mammalian Cells

Two cell lines were selected for conducting mammalian cell culture:

- Saos-2 (ATCC, Manassas, VA, USA)—treated as a stable line of osteoblast-like human cells,
- EA.hy926 (ATCC, Manassas, USA)—hybrid of human endothelial cells isolated from umbilical vein (HUVEC) and human lung cancer cells (A549).

Saos-2 cells were cultured in McCoy’s 5A medium supplemented with 15% fetal bovine serum. As a culture medium for EA.hy926 cells, Dulbecco’s modified Eagle’s medium was used, to which 10% of fetal bovine serum was added. The media for both lines also contained antibiotics: penicillin and streptomycin.

#### 2.6.1. Cells Metabolic Activity

To assess cells proliferation ability, a colorimetric assay (ATCC, Manassas, VA, USA) was used based on the evaluation of mitochondrial dehydrogenase activity to transform water-soluble tetrazole salt (2,3-bis(2methoxy-4-nitro-5-sulfophenyl)-2H-tetrazolo-5-carboxanilide-XTT) to a soluble formazan product. Three samples were analyzed for each tested material. They were placed individually in 12-well plates (Corning costar) with 2 mL of culture medium. The seeded density in case of each cell line was 6 × 10^4^ cells/cm^2^. Plates were incubated for 48 h at 37 °C in an atmosphere containing 5% CO_2_. After this time, medium was removed from each well and all adhered cells (both from the samples and walls of the container) were detached using trypsin. The XTT test was carried out with 100 μL of suspension containing approximately 5 × 10^3^ of previously obtained cells in culture medium and 0.5 μL of XTT reaction mixture. For the sake of control preparation, the procedure was repeated but without samples in the well). After 4 h of incubation, the absorbance of the mixture was measured at 450 nm and 620 nm. For this purpose, a microplate spectrophotometer (Multiskan GO, Thermo Scientific, Waltham, MA, USA) was used. Cell viability was determined using the following equation:

V (%) = (A/AC) × 100%

where: V-cell viability expressed in % of control; A-specific absorbance of the tested mixture; AC-specific absorbance of the control mixture not containing sample.

#### 2.6.2. Cell Viability

To assess cell viability, the LIVE/DEAD test was used using an InCell Analyzer 2000 automated microscope (GE Healthcare, Chicago, IL, USA). Cells of examined cell line were seeded on the surface of tested materials at constant amount of about 12 × 10^4^ per well in 2 mL of medium suitable for a given cell line and then cultured for 48 h under standard conditions. As a control, a well with no sample was used. Before observations under a fluorescent microscope, the medium was removed and the cells were washed with PBS solution. The samples were then incubated for 15 min at room temperature in a balanced Hank salt solution containing a mixture of fluorescent dyes: Hoechst 33342 (Molecular Probes, Eugene, OR, USA), calcein AM (Santa Cruz Biotechnology, Dallas, TX, USA), and propidium iodide (Molecular Probes).

The obtained images were analyzed using InCell Analyzer (GE Healthcare) software. All cells were divided into two subpopulations, i.e., live (stained with calcein-AM—gives green color) and dead (stained with propidium iodide—red color). The total number of cells was determined based on blue fluorescence of Hoechst 33342.

### 2.7. Statistical Analysis

The statistical analysis of the obtained results of biological response was performed using the one-way analysis of variance (ANOVA) with a significance level of *p* = 0.075. Only the total number of cells was taken into account. Each pair of statistically significant results were denoted on graphs by one of the following symbols: *; □; ○; ◊; ∆; and +.

## 3. Results

### 3.1. Surface Characterization

**Figure 1:**
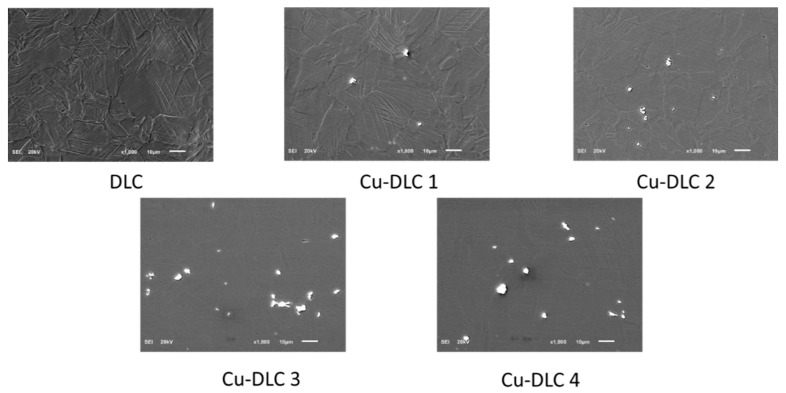
Exemplary SEM pictures of Cu-DLC (diamond-like carbon) coatings deposited on AISI 316 LVM (magnification × 1000, secondary electron image (SEI) mode).

**Figure 2:**
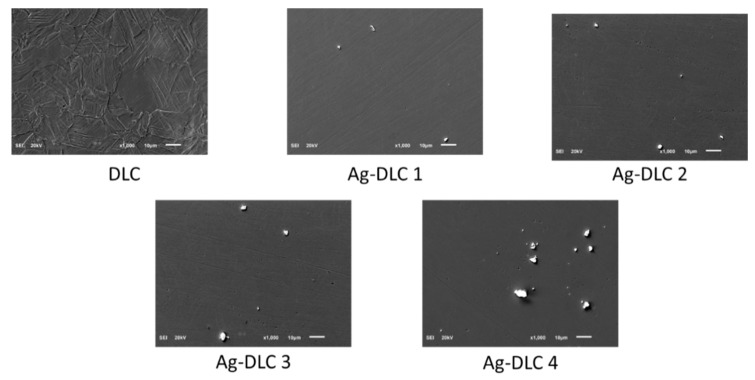
Exemplary SEM pictures of Ag-DLC coatings deposited on AISI 316 LVM (magnification × 1000, SEI mode).

SEM analysis performed in SEI mode proves that synthetized coatings were continuous, free of local delamination or defects. Due to the coating thickness of only up to 200 nm, each time, pictures (see Figure 1 and Figure 2) revealed the structure of the given substrate material. Both in case of Ag-DLC and Cu-DLC, unevenly distributed agglomerates were visible. They were identified by spot-EDS analysis as additive material. The number and size of the agglomerates was increasing with increasing quantity of admixture material in the coating (specified by XPS analysis). EDS analysis performed on areas without agglomerates confirmed the presence of admixtures also in the carbon matrix.

Quantitative surface chemical analysis performed by means of XPS showed that magnetron sputtering of only graphite targets result in coatings without trace amount of silver or copper. The composition of DLC coating deposited on various substrates was similar: about 81.5 at.% of carbon and 18.5 at.% of oxygen (see Table 1).

For the X-DLC coatings, the presence of admixture was proven by the presence of their characteristic peaks: Cu-2p_3/2_ at approximately 933.7 eV or Ag-3d_5/2_ at approximately 368.3 eV and Ag 3d_3/2_ at approximately 374.3 eV. The amount of silver and copper in all the examined samples was summarized in Table 2.

**Figure 3:**
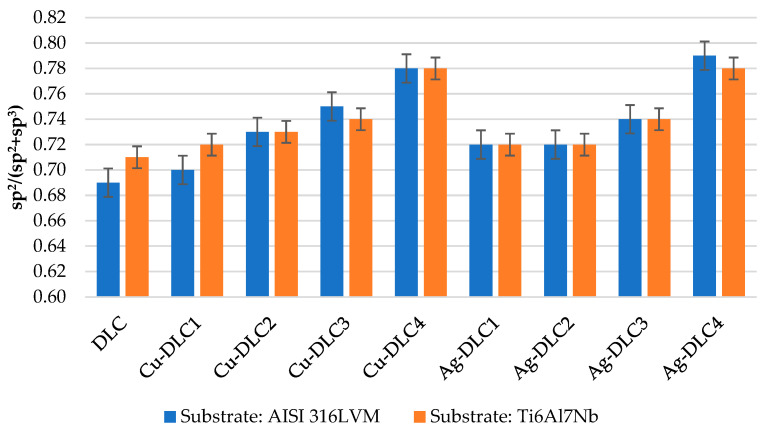
The sp^2^/(sp^2^ + sp^3^) ratio calculated on the basis of deconvolution of C1s peak.

The XPS analysis was also used to verify types of chemical bonds between the coating elements. By the deconvolution of C1s peak (284.6 eV), it was possible to determine the amount of sp^2^ (284.5 eV) and sp^3^ (285.3 eV) carbon–carbon bonds, as well as those existing between carbon and oxygen (286.1 eV and 288.0 eV). As presented in Table 3, the sputtering of Cu or Ag source resulted in an increased amount of sp^2^ hybridized bonds for all analyzed coatings. The ongoing graphitization is also evidenced by the growing sp^2^/(sp^2^ +sp^3^) ratio (see Figure 3). For low content of admixtures (below 1%), the increase in the number of sp^2^ hybridized bonds was not accompanied by a decrease in the number of sp^3^ hybridized bonds. A silver amount of several percent can lead to about 23% depletion of that parameter, while in the case of copper, it was almost 28%. The percentage of C–O and C=O bonds was similar for all the examined coatings, which was about 8.3% and 7.3%, respectively.

The bond energies for metallic silver and its oxidized forms are defined by the following intervals: 367.9–368.7 eV for metallic silver—Ag^0^; 367.6–368.5 eV for Ag_2_O; and 367.3–368.1 eV for AgO [34,35,36]. For all the examined Ag-DLC samples, Ag 3d_5/2_ maxima were located in the range of 368.3–368.6 eV, which proves the presence of silver at 0 and +1 oxidative state.

**Figure 4:**
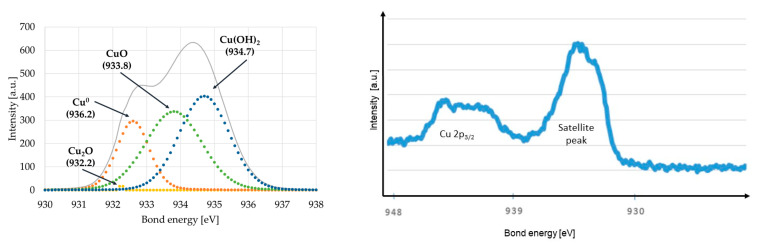
Curve fitting of Cu 2p_3/2_ XPS spectra—example for Cu-DLC1 coating deposited on AISI 316LVM (**left**). The Cu 2p_3/2_ peak and the satellite peak for Cu-DLC4 coating deposited on Ti6Al7Nb alloy (**right**).

Bond analysis of Cu-DLC samples was conducted according to following bond energies: Cu^0^—932.6 eV; Cu_2_O—932.2 eV; CuO—933.8 eV; Cu(OH)_2_—934.7 eV [37] (see Figure 4 left). As presented in Table 4, in all the coatings, only Cu_2_O bonds did not exceed 2%. The amount of metallic copper was in the range of 20.3% to 27.8%. For the highest content of copper in the carbon matrix, the increase of the percentage of Cu(OH)_2_ related with decrease of CuO bonds was visible. The additional evidence of the presence of copper at the oxidation level +2 in all the coatings was a characteristic satellite peak (i.e., shake-up peak) occurring around 944 eV (Figure 4 right).

**Figure 5:**
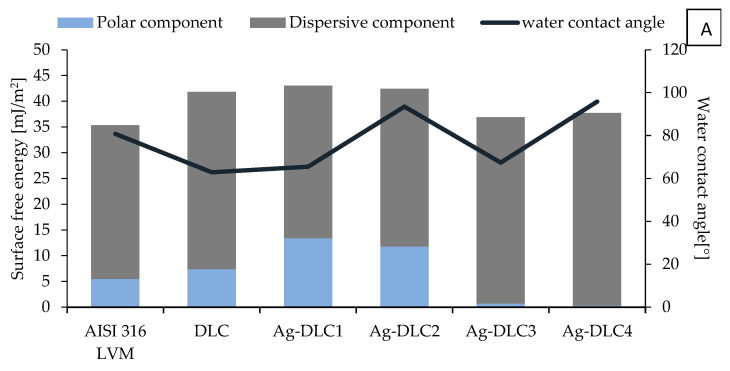

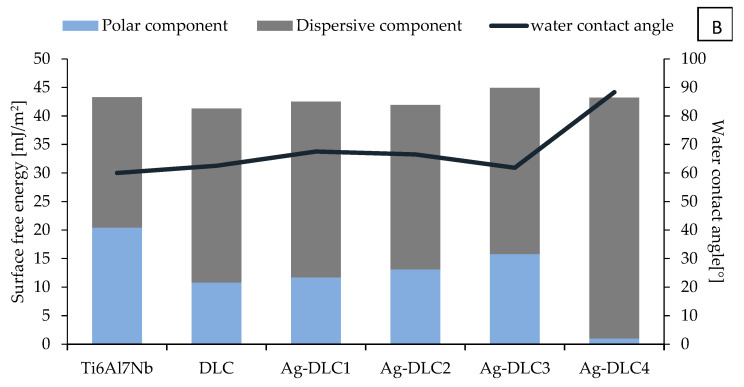
Surface energy components and contact angle for water of Ag-DLC coatings on AISI 316LVM (**A**) and Ti6Al7Nb (**B**) substrates.

**Figure 6:**
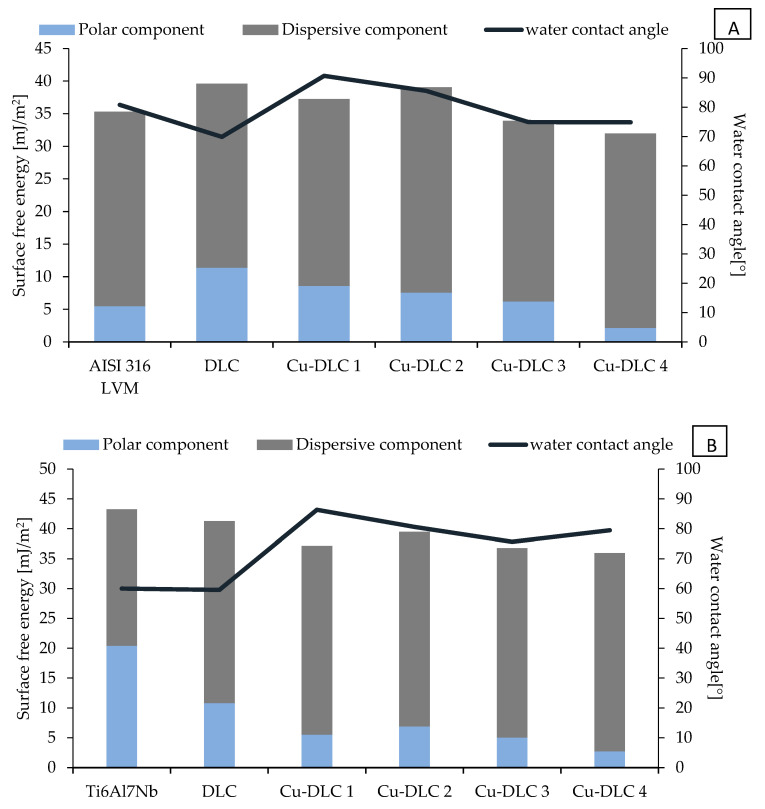
Surface energy components and contact angle for water of Cu-DLC coatings on AISI 316LVM (**A**) and Ti6Al7Nb (**B**) substrates.

Both total SFE (polar and dispersive components) and values of contact angle for water are presented in Figure 5 and Figure 6. The hydrophobicity of the coatings increased with the addition of elements of admixture. SFE of Ag-DLC coatings was at a similar level, ranging from 35 to 42 to mJ/m^2^. For silver content of about 7 at.% and higher, the depletion of polar component to the level of 1 mJ/m^2^ was observed.

Considering Cu-DLC coatings, for all the samples, the value of SFE was lower than in the case of un-doped DLC at the given substrate material. The polar component of surface energy decreases with higher amounts of copper in the carbon matrix. Despite a slight increase in the dispersion component, the SFE value also decreases for coatings containing increasing Cu content.

### 3.2. Assessment of Microbial Colonization

**Figure 7:**
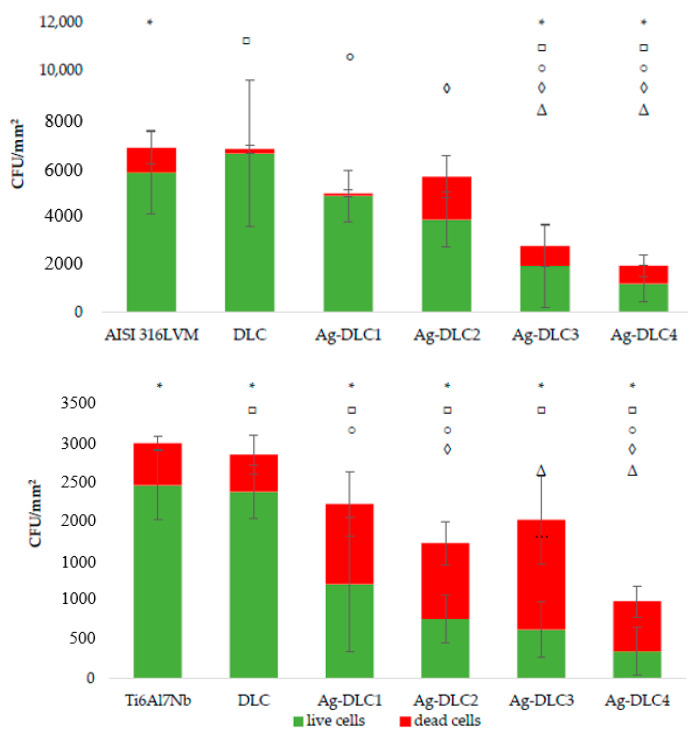
Assessment of microbial colonization of *Escherichia coli* on Ag-DLC coatings deposited on AISI 316LVM (**top**) and Ti6Al7Nb (**bottom**).

**Figure 8:**
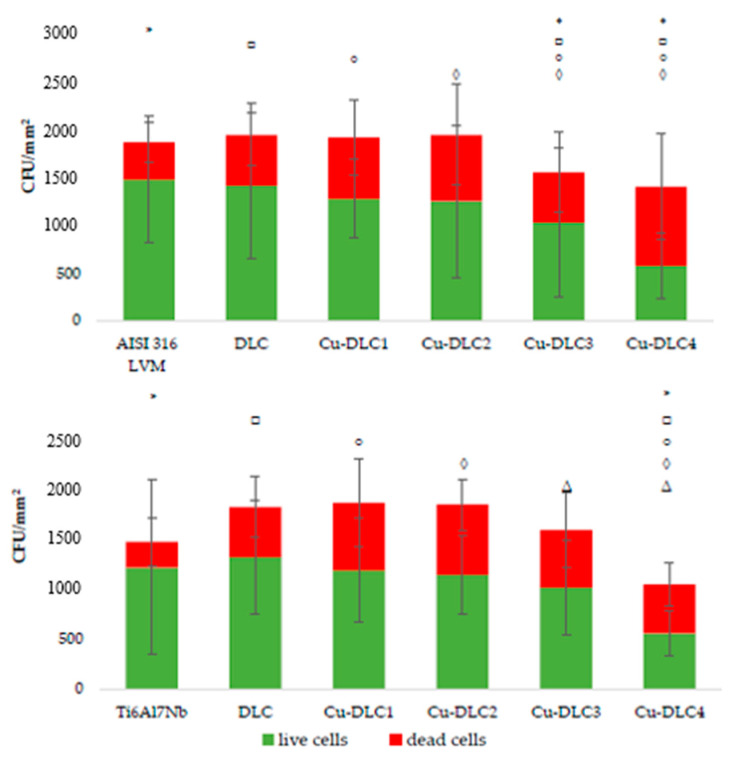
Assessment of microbial colonization of *E. coli* on Cu-DLC coatings deposited on AISI 316LVM (**top**) and Ti6Al7Nb (**bottom**).

Effect of various amounts of Ag and Cu in a carbon matrix on biofilm formation is shown in Figure 7 and Figure 8. The addition of even the smallest examined amount of silver (about 0.65 at.% and 0.49 at.%, respectively, of substrate material) to the carbon matrix resulted in the reduction of living microbial cells on the examined surfaces. Furthermore, only except from Ag-DLC1 deposited on stainless steel, the addition of that element caused a significant increase in the number of dead cells. In the case of total microbial cell count, observable was a decreasing tendency with an increasing amount of silver. In a few cases (Ag-DLC2 deposited on AISI 316LVM and Ag-DLC3 deposited on TI6Al7Nb), such a trend was distorted by a significantly higher amount of dead cells. Regardless of the substrate used, the smallest total number of microbial cells was adhered to the coatings with the maximum concentration of silver that was tested. For the AISI 316 LVM, the total reduction of microbial colonization equaled to 71% of the value for DLC coating, while for the titanium alloy, it was about 66%.

Regardless of the substrate used, along with the increase of copper content in the carbon matrix, there was a gradual decrease in both the average number of bacterial cells and living cells adhering to the tested surfaces. The reduction in the number of viable *E. coli* cells for Cu-DLC 4, in comparison to un-doped DLC, was statistically significant and equal to approximately 45% (48% for coatings deposited on AISI 316 LVM and 24 for Ti6Al7Nb substrate). Additionally, the ratio of live to dead cells was the lowest for the Cu-DLC4 coating, regardless of the applied substrate. In all the examined cases, the addition of copper increased the number of dead cells. However, there was no clear relationship in the effect exerted by this admixture on the number of dead cells.

### 3.3. In Vitro Evaluation with Mammalian Cells (XTT Test Results)

**Figure 9:**
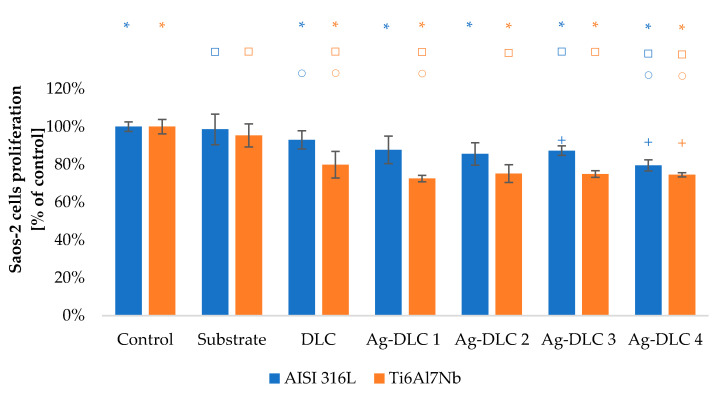
Assessment of proliferation of Saos-2 cells in contact with Ag-DLC coatings. Results of XTT examination.

**Figure 10:**
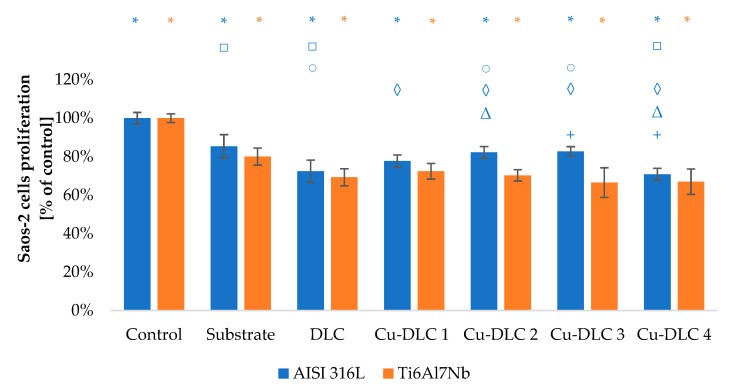
Assessment of proliferation of Saos-2 cells in contact with Cu-DLC coatings. Results of XTT examination.

**Figure 11:**
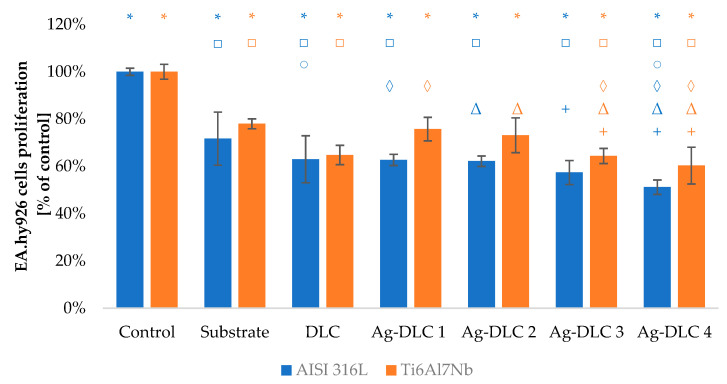
Assessment of proliferation of EA.hy926 cells in contact with Ag-DLC coatings. Results of XTT test examination.

**Figure 12:**
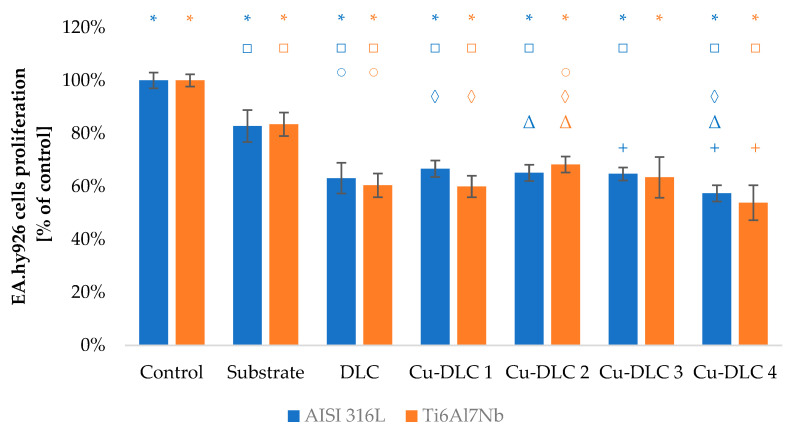
Assessment of proliferation of EA.hy926 cells in contact with Cu-DLC coatings. Results of XTT test examination.

The viability of Saos-2 osteoblasts due to the contact with carbon coatings was at least on the level of 70% of the control sample (Figure 9 and Figure 10). In the case of EA.hy926 cells, that value was only 65% (Figure 11 and Figure 12). Introduction of admixture to the carbon coatings had a greater and more diverse effect on the proliferation of EA.hy926 endothelial cells than on Saos-2 osteoblast cells.

In all analyzed Ag-DLC coatings, the proliferation level of osteoblast cells was close to the values obtained by un-doped coatings. The maximum difference of approximately 15% for a steel substrate and 10% for a titanium substrate was obtained, respectively, for coatings Ag-DLC3 and Ag-DLC1.

The addition of a small amount of copper to the carbon matrix (up to 4.74 at.%) improved proliferation ability of Saos-2 cells. Such effect was visible for samples Cu-DLC1, Cu-DLC2, and Cu-DLC3 but only for AISI 316 LVM substrate. The increase was on the level of 5–10% of control. In the case of titanium alloy as a substrate material, the statistically significant difference appeared only between samples DLC and Cu-DLC 1—an improvement of about 3%. No positive effect or even slight decrease of proliferation level in comparison to the un-doped sample was visible for Cu-DLC4 deposited on both substrate materials.

Assessment of proliferation ability of EA.hy926 cells showed almost a depletion of cell viability with the increasing amount of silver in doped coatings. The depletion reached for Ag-DLC4 approximately 12% and 10%, respectively, for AISI 316LVM and TI6Al7Nb substrates. In the case of coatings deposited on stainless steel, the proliferation deterioration occurred even with the lowest amounts of admixture. For the second substrate, an improvement of viability of about 5% comparing to the control sample was observable for Ag-DLC1 and Ag-DLC2.

The presence of the highest examined amount of copper in carbon matrix resulted in a decrease in EA.hy926 cell viability by about 5% compared to DLC. Such a difference was statistically significant for titanium substrate material. For a lower amount of that element, cell viability remained at an almost constant level for AISI 316 LVM substrate or even slightly elevated for the second substrate material. The highest viability was observed for Cu-DLC2 coating deposited on titanium alloy—its level was about 8% more than for pure carbon coating. Except for direct assessment of proliferation taking into account the total number of cells, in all the examined cases (substrates, un-doped, and doped coatings), the division into dead and leaving cells was conducted. In all the cases, percentage of dead cells did not exceed 15%.

**Figure 13:**
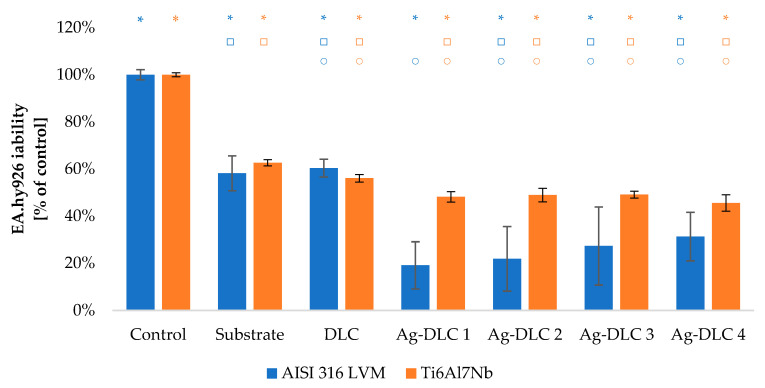
Evaluation of viability of EA.hy926 cell on the surface of Ag-DLC coatings deposited on AISI 316LVM and Ti6Al7Nb (live/dead test). Total number of cells expressed as a percentage of control.

**Figure 14:**
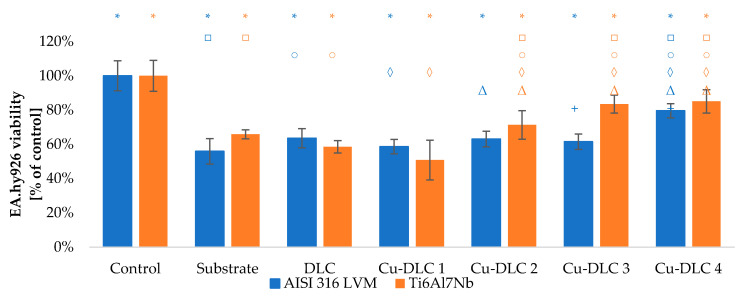
Evaluation of viability of EA.hy926 cell on the surface of Cu-DLC coatings deposited on AISI 316LVM and Ti6Al7Nb (live/dead test). Total number of cells expressed as a percentage of control.

**Figure 15:**
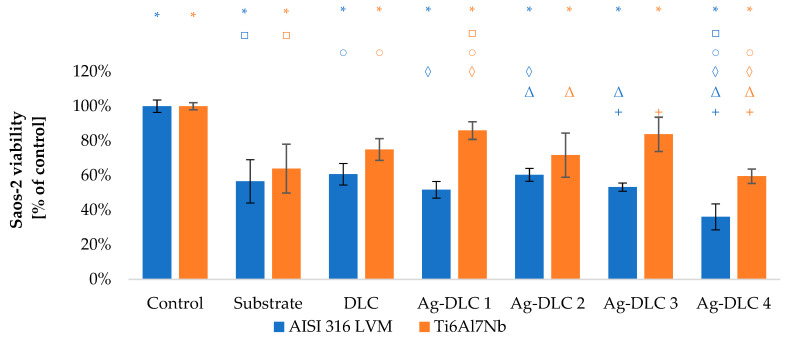
Evaluation of viability of Saos-2 cell on the surface of Ag-DLC coatings deposited on AISI 316LVM and Ti6Al7Nb (live/dead test). Total number of cells expressed as a percentage of control.

**Figure 16:**
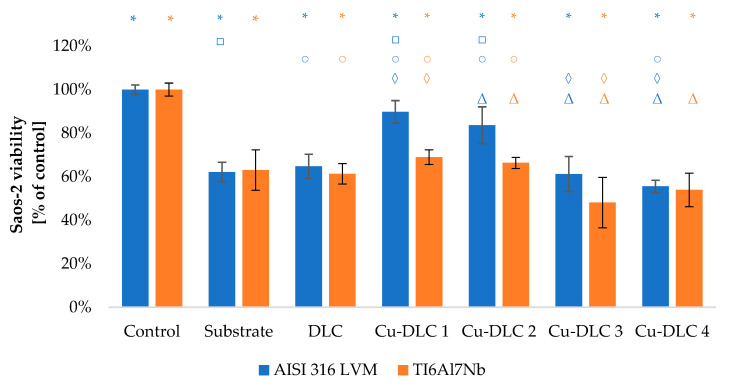
Evaluation of viability of Saos-2 cell on the surface of Cu-DLC coatings deposited on AISI 316LVM and Ti6Al7Nb (live/dead test). Total number of cells expressed as a percentage of control.

The viability of both Saos-2 and of EA.hy926 cells on the substrate material, as well as un-doped DLC coating, was equal to 60% of control sample (Figure 13, Figure 14, Figure 15 and Figure 16). There are no linear tendencies between the amount of admixture and the level of viability in the case of osteoblast cells. 

Viability of Saos-2 observed on coatings doped with silver and deposited on AISI 316 LVM was lower than in the case of Ti6Al7Nb substrate. However, increased viability for coatings deposited on titanium alloy was concomitant with a higher percentage of observed dead cells (in range from 6 to 11% of control). The only exception in that manner was sample Ag-DLC2, for which the level of dead cells was similar on samples on both substrate materials. However, regardless of the substrate material, results obtained for Ag-DLC4 showed a deterioration of the biological response. It was visible in the reduction of viability by approximately 35% and 25%, respectively, for stainless steel and titanium alloy. Furthermore, the amount of dead cells reached 5% for that sample.

Among examined samples, a lower copper content in deposited coatings (Cu-DLC1 and Cu-DLC2) increased osteoblast viability, but the effect had different intensity depending on the substrate material. In the case of Ti6Al7Nb, the level of about 68% was achieved, while for AISI 316 LVM, that value was much closer to the control—approximately 83%. For samples with a higher content of copper, the effect is opposite, and for Cu-DLC4, the level of viability was depleted to approximately 55%. The percentage of dead Saos-2 cells on Cu-DLC coatings was higher than for un-doped coating regardless of the substrate. However, it remains within 6% of the control. This was also the level that occurred for an AISI 316LVM substrate.

The presence of silver in carbon coatings had a negative effect on endothelial cell viability. Regardless of the substrate on which the tested coatings were deposited, the percentage of the total number of EA.hy926 cells was lower than the value obtained for the un-doped coating. Ag-DLC coatings achieved a viability of at most 31.5% of the control for a stainless steel substrate, and at most 49.6% of the control for a Ti6Al7Nb substrate. The reduction of EA.hy926 cell viability on Ag-DLC coatings was not associated with an increase in the number of dead cells.

The highest examined content of copper resulted in an increase in the number of live endothelial cells by approximately 16% compared to the un-doped DLC coating. Such a value is similar to that obtained for control sample. Furthermore, the number of dead EA.hy926 cells was the lowest for Cu-DLC4. In the case of titanium substrate, the mentioned improvement of viability was a gradual change with increasing Cu concentration. For coatings on AISI 316 LVM substrate, the value for DLC, Cu-DLC1, Cu-DLC2, and Cu-DLC3 was on a similar level. 

## 4. Discussion

The percentage of admixtures in the deposited coatings did not exceed 7 at.% and 15 at.%, respectively, for copper and silver. For each admixture element and metal substrate used, samples containing less than 1% at. of added elements, as well as a few percent, were prepared. Currently, research conducted on Cu-DLC and Ag-DLC coatings covers a similar range of content of the mentioned elements, especially in the case of biological research [17,38,39,40].

Synthetized coatings contained a significant amount of oxygen. The presence of that element was connected with high reactivity of the surface after the process of deposition that results in the saturation of free carbon bonds with atmospheric gases and water vapor. Literature describes even up to 20 at.% of oxygen on surfaces of doped DLC [41].

As shown by XPS analyses, even small amounts (less than 1 at.%) of silver and copper present in carbon coating was associated with an increase in sp^2^ hybridized bonds by about 5%, while maintaining the amount of sp^3^ hybridized bonds at a level similar to un-doped coatings (between 23% and 25%). In the case of the maximum analyzed amount of both silver and copper in carbon coatings, a decrease in the number of bonds with sp^3^ hybridization from 4% to 8% was visible. This has probably the effect on reduction of the hardness of the produced coatings. The Cu-DLC coatings with intermediate contents of examined elements were characterized by the size of the sp^2^/(sp^2^ + sp^3^) ratio at a similar level. In the case of Ag-DLC coatings, a gradual decrease in the content of sp^3^ hybridization bonds and an increase in the amount of sp^2^ hybridization bonds with an increase in the amount of admixture in the coatings were observed. Regardless of the type and amount of the additive, the content of sp^2^ hybridization bonds did not exceed 70%.

According to the results presented by research from all over the world, doping of carbon coatings with silver and copper leads to an increase in the surface water contact angle, which is associated with the more hydrophobic nature of the synthetized material-due to presence of the admixture [42,43]. There are two main factors that affect the decrease in SFE of doped coating. First of all, coatings are affected by graphitization [44], and regions rich in sp^2^ hybridization bonds can increase surface hydrophobicity by reducing the amount of free bonds on the surface. An admixture in metallic form can also affect the SFE [45]. Although the results of XPS analysis indicated a high degree of metal oxidation present in the manufactured coatings, the amount of those elements in metallic state combined with surface graphitization probably led to an overall reduction of the polar component of surface energy observable both for Cu-DLC in comparison to un-doped coatings and for Ag-DLC samples with over 3% of silver.

In the case of biological properties, the dominant effect (both for microbial and mammalian cells) of chemical composition over the wettability is visible in all the doped coatings. The bactericidal or bacteriostatic effect of DLC coatings was evaluated by characterization of the bacterial cells in contact with examined surfaces.

*E. coli* is a microorganism commonly used to conduct microbiological research in the field of genetics (e.g., DNA replication analysis [46]), biotechnology [47], as well as biomedical engineering [48] or material engineering [28]. The *E. coli* strain is responsible for a variety of peri-implant infections. *Escherichia coli* is one of the leading causes of Gram-negative infections in orthopedic implants, but little is known about the pathogenicity of this species in the context of post-implantation infections. Moreover, as numerous publications show, *E. coli* does not show the same infection-spreading strategies as, for example, *S. aureus* against, inter alia, osteoblasts [49]. The potential of all analyzed doped carbon coatings to reduce the total number of *E. coli* cells developing on their surface was confirmed. However, it should be noted that in the case of Cu-DLC coatings, it is necessary to use at least a few percent of the admixture content in order to reduce microbial colonization, while for silver, changes occur even for the content below 1 at.%. Use of Ag-DLC coatings enables to reduce the amount of bacteria by even 70%. Despite the proven bactericidal properties, the use of Ag-DLC coatings in biomedical applications may involve negative impact on the condition of the surrounding tissues.

In the case of copper-doped coatings, the lowest total number of all microorganisms (including living and dead cells) and a high percentage of dead cells may indicate the bactericidal nature of only Cu-DLC4 coatings regardless of substrate material. Publications describing almost complete eradication of tested microorganism focused on copper content above 20% [17,48]. A slight decrease in the number of live bacteria, in the case of other samples containing copper, indicates a low bacteriostatic effect of Cu at contents lower than 6.85 at.% for AISI 316LVM substrate and 5.99 at.% for Ti6Al7Nb substrate. The total number of cells higher than for substrate material in the case of DLC, Cu-DLC1, Cu-DLC2, Cu-DLC3 deposited on Ti6Al7Nb shows that copper admixture should be avoided in such connection for potential implantation purposes.

Silver in carbon coatings, even in quantities below 1%, resulted in a much higher decrease in the number of affected *E. coli* cells than in analogous amounts of copper. The presence of silver in DLC coatings was associated with a significant increase in the number of dead cells, which correlates with the bactericidal nature confirmed for this admixture [50,51]. The use of the maximum tested amount of Ag in Ag-DLC allowed for the reduction of the total amount of microbial cells to 30% of their amount on un-doped DLC. Those results are comparable with the reports of Goudouri et al. [52], who proved a stronger influence of Ag than Cu on the limitation of microbial colonization of the surface. 

To evaluate the biological response in terms of safety of examined materials, the microbiological test was compared with the response of mammalian cells. Research on endothelial (EA.hy926) and osteoblast (Saos-2) cells were performed in two complementary ways: evaluation of their proliferation ability and viability after contact with examined materials. All tested samples—basic metallic substrates, reference coating DLC, and doped coatings—did not cause extensive cell death (more than 10%), nor excessive proliferation (the results higher than the control might indicate the carcinogenic nature of the coatings). Although the use of the osteoblast cell line is self-explanatory for research on coatings for orthopedic implants, the selection of endothelial cell line is worth a comment. EA.hy926 cells are commonly used cells in biological investigation practice. It needs to be noted that a prolonged contact of endothelial cells with biomaterial may be of key importance for the course of many biological processes taking place within the implantation area. The response of endothelial cells to such contact with abiotic, artificial surface, can be a very important factor in inflammation, blood clotting, angiogenesis, and many other processes in which endothelial cells are involved. In the case of, for example, vessel reconstruction or heart surgery, the endothelial cells remain in direct contact with the implant surface. Studies on the interactions of the endothelial cells with the surface of biomaterial are also very important in the field of biomaterial production [53].

The effect of copper on the osteoblast cell line is highly dependent on dose of that element. The presence of small amounts of copper (Cu-DLC1 and Cu-DLC2) leads to the increase of cells proliferation. In the case of AISI 316 LVM substrate, results were similar as in the case of control, so for cells with no contact with tested material. Literature reports show that low levels of copper in physiological fluids can promote the transformation of mesenchymal stem cells into osteoblasts [54,55] and stimulate proliferation of osteogenic cells [30]. It seems that a similar effect appears for Cu-DLC coatings. As copper content in carbon coatings reaches 2.5–5 at.%, proliferation of Saos-2 returns to the level of un-doped coatings or even slightly deteriorates. These results correlate with the results of Zhang et al. [56] for porous TiO_2_ doped with copper nano-particles. In that study, the presence of 1.3 at.% copper promoted the proliferation and adhesion of osteoblasts, but the higher amount—2.76 at.%—induced cytotoxicity of the MC3T3-E1 cell line. Hang et al. [57], dealing with TiO_2_ nanotubes with the addition of CuO, obtained the limiting content of admixture (minimal level which does not induce negative effect on osteoblasts), equal to 1.01 at.%. On the other hand, according to some reports already, 0.8 wt. CuO is harmful to bone regeneration [58]. Changes in osteoblast proliferation also reflect their viability. Cu-DLC coatings increase the viability of this cell line; however, the content of this element must be limited. For the highest content of tested admixtures, the positive effect of copper is probably overpowered by its toxic nature (confirmed, e.g., in microbiological tests). Such negative or even cytotoxic effect of high concentration of Cu^2+^ ions on Saos-2 cells was proven by Huang et al. [59].

Analysis of the proliferation of EA.hy926 cells in contact with doped DLC coating showed a positive effect of copper in carbon matrix. The character of that improvement was variable depending on the type of substrate material. For stainless steel substrate, proliferation increases only at the highest concentration of this element, while for a titanium alloy substrate, the improvement was gradual. Such an effect is probably related to the presence of copper in the second oxidation state. XPS analysis showed that the amount of CuO and Cu(OH)_2_ bonds was also changing in the same manner. In vitro studies have shown a positive effect of the presence of copper ions on proliferation of endothelial cells and stimulation of expression of genes important in wound healing [31,60]. Copper also influences angiogenesis (blood vessel formation) by stimulation of vascular endothelial growth factor [61,62]. Unfortunately, the improvement in endothelial cell proliferation for Cu-DLC4 samples is associated with a reduction in their viability. Similar to the effect of high copper content on osteoblast cells, it may be associated with the negative (toxic) nature of this admixture, which in this amount, negatively affects the metabolic processes of cells.

The use of copper in carbon coatings in amounts improving bactericidal properties (about 5–7 at.%) eliminates the positive effect of this admixture in the case of contact of the coatings with both endothelial cells and osteoblasts. Although Cu-DLC coatings improve EA.hy926 cell proliferation at high amounts of copper, and Saos-2 at low admixture levels, the overall reduction of viability (for both cell lines) below the level of safety index at the level of 70% for XTT test (that is in line with standard EN 10933-5) is alarming.

The amount of silver that proves bactericidal effect of Ag-DLC coatings may have negative effect on osteoblasts. Alberts et al. [63] determined that the cytotoxicity of Ag^+^ ions released from silver nano-particles already takes place at a concentration of 2–4 times lower than for bactericidal properties. This means that even Ag-DLC1 coatings, which caused a decrease in the number of targeted *E. coli* bacteria, could reduce Saos-2 cell proliferation. Nevertheless, such an effect was visible for the highest tested silver content. In case of Ag-DLC4 samples, a significant decrease in the proliferation of Saos-2 occurred. Similar results were obtained with MC3T3 cells, where coating containing 5.5 at.% Ag decreased their number in the first days of incubation [64]. The reduction of proliferation had no negative effect on the viability of the Saos-2 cell line.

Ag-DLC samples were characterized by a decrease in both endothelial cell proliferation viability. That effect was predominant for coatings deposited on stainless steel. In the literature, the negative effect of silver on endothelial cells was determined primarily for silver nano-particles [65]. Additionally, studies conducted for various cell lines and silver doped calcium phosphate powder in amounts up to 3% by weight showed a decrease in the viability of cells belonging to the HUVEC (endothelium) and V79 379 (fibroblasts) lines [66].

The safety index for viability shows that the addition of silver in coatings developed for contact with endothelial cells is considerable only for concentrations lower than 1 at.% and Ti6Al7Nb substrate. Nevertheless, the proliferation of this cell line has been decreasing even for such small amounts of silver. Ag-containing coatings did not adversely affect the Sao2-2 line with the exception of a reduction in proliferation by Ag-DLC4 samples.

## 5. Conclusions

Although silver in the produced doped DLC coatings occurs primarily in the metallic, while copper in the oxidized form, in the case of introduction of both elements, the increasing number of agglomerates is visible on the surfaces for higher admixture content. The surface properties are affected also by graphitization of DLC because of introduction of additives their amount highly affects biological properties.

Carbon coatings with the addition of copper or silver allow the reduction of the total number of *E. coli* cells developing on their surface. Only about 10 at.% and a higher content of silver allows the reduction of bacteria by about 70%. Ag-DLC coatings have a high potential as a bactericidal material, but their use as a coating for orthopedical implants is associated with a decrease in the biological response of endothelial cells. The amounts of copper necessary to induce the bactericidal properties (about 5–7 at.%) of carbon-based coatings do not allow their use for implantology because of of low viability level of both cells cultures, the Saos-2 and EA.hy926 lines, assessed by XTT test. Further studies on the addition of copper should involve the range of at least 5–7 at.% so as to obtain bactericidal properties while maintaining the potential of these coatings to improve the positive effect on the development of both endothelial cells and osteoblasts. For the preparation of bactericidal orthopedical implants, the incorporation of silver in the amounts in the range of 1 to 10 at.% may ensure both a reduction of bacteria and positive effect on proliferation and viability of osteoblasts.

## Figures and Tables

**Table 1:** Surface chemical composition of non-doped carbon coatings—results of XPS examination.

Coating	Chemical Composition	
	O (at.%)	C (at.%)
DLC substrate: AISI 316LVM	18.15 ± 0.37	81.85 ± 0.40
DLC substrate: Ti6Al7Nb	18.44 ± 0.11	81.56 ± 0.15

**Table 2:** Surface amount of admixtures in X-DLC (X stands for additive element (Ag or Cu)) coating evaluated by means of XPS examination.

Ag-DLC		Cu-DLC	
Substrate: AISI 316 LVM		Substrate: AISI 316 LVM	
Sample ID	Ag (at.%)	Sample ID	Cu (at.%)
DLC	0.00	DLC	0.00
Ag-DLC1	0.65 ± 0.10	Cu-DLC1	0.48 ± 0.08
Ag-DLC2	0.86 ± 0.09	Cu-DLC2	1.23 ± 0.05
Ag-DLC3	6.99 ± 0.70	Cu-DLC3	4.74 ± 0.46
Ag-DLC4	9.77 ± 0.98	Cu-DLC4	6.85 ± 0.16
**Substrate: Ti6Al7Nb**		**Substrate: Ti6Al7Nb**	
**Sample ID**	**Ag (at.%)**	**Sample ID**	**Cu (at.%)**
DLC	0.00	DLC	0.00
Ag-DLC1	0.49 ± 0.07	Cu-DLC1	0.98 ± 0.03
Ag-DLC2	0.82 ± 0.07	Cu-DLC2	1.38 ± 0.09
Ag-DLC3	2.58 ± 0.23	Cu-DLC3	2.60 ± 0.40
Ag-DLC4	13.38 ± 1.20	Cu-DLC4	5.99 ± 0.20

**Table 3:** Analysis of C1s peak—percentage content of bonds that were present in X-DLC coatings.

	Substrate: AISI 316LVM				Substrate: Ti6Al7Nb			
	sp^2^	sp^3^	C–O	C=O	sp^2^	sp^3^	C–O	C=O
DLC	56.4 ± 1.9	24.9 ± 1.1	12.5 ± 0.2	6.3 ± 0.4	58.7 ± 0.7	23.3 ± 0.7	11.8 ± 0.1	6.3 ± 0.1
Cu-DLC1	61.7 ± 0.2	24.6 ± 1.1	7.7 ± 0.8	5.9 ± 0.5	61.7 ± 1.0	24.4 ± 1.0	5.6 ± 0.7	8.3 ± 0.5
Cu-DLC2	62.1 ± 1.0	24.6 ± 1.1	6.1 ± 0.5	7.2 ± 0.2	62.5 ± 0.7	24.0 ± 1.2	6.2 ± 0.5	7.2 ± 0.4
Cu-DLC3	63.8 ± 1.0	23.0 ± 0.6	7.1 ± 1.1	6.1 ± 1.0	63.5 ± 0.8	22.8 ± 1.0	8.3 ± 0.5	5.3 ± 0.3
Cu-DLC4	64.1 ± 2.6	16.6 ± 2.5	7.4 ± 0.5	11.9 ± 0.6	65.7 ± 0.8	18.6 ± 0.8	8.2 ± 0.8	7.5 ± 0.7
Ag-DLC1	60.3 ± 0.4	25.7 ± 3.0	7.8 ± 2.8	6.2 ± 0.4	61.4 ± 0.1	24.3 ± 0.3	7.6 ± 0.3	6.8 ± 0.5
Ag-DLC2	62.8 ± 1.3	23.3 ± 2.8	12.4 ± 2.0	1.4 ± 3.0	62.4 ± 2.3	23.6 ± 1.5	8.1 ± 0.7	5.8 ± 0.6
Ag-DLC3	63.6 ± 0.5	21.1 ± 0.8	9.0 ± 0.4	6.2 ± 0.4	64.0 ± 0.7	23.0 ± 0.4	4.6 ± 0.2	8.5 ± 0.3
Ag-DLC4	69.4 ± 2.1	19.0 ± 3.0	6.5 ± 1.5	5.1 ± 0.9	67.7 ± 1.0	36.2 ± 1.0	5.3 ± 0.6	7.5 ± 0.7

**Table 4:** Deconvolution of Cu 2p_3/2_ peak obtained in the XPS analysis.

	Cu 2p_3/2_ (eV)	Bond Type	AISI 316LVM	Ti6Al7Nb
			Share of Bonds	Share of Bonds
Cu-DLC1	932.2	Cu_2_O	0.1% ± 0.1%	0.4% ± 0.3%
	932.6	Cu^0^	20.3% ± 1.0%	27.8% ± 0.9%
	933.8	CuO	38.8% ± 0.9%	38.4% ± 1.0%
	934.7	Cu(OH)_2_	40.8% ± 1.2%	33.4% ± 1.1%
Cu-DLC2	932.2	Cu_2_O	0.1% ± 0.1%	0.3% ± 0.1%
	932.6	Cu^0^	22.1% ± 0.9%	24.7% ± 0.5%
	933.8	CuO	39.2% ± 1.0%	37.0% ± 0.6%
	934.7	Cu(OH)_2_	38.6% ± 0.8%	38.1% ± 1.0%
Cu-DLC3	932.2	Cu_2_O	1.4% ± 0.9%	0.8% ± 0.9%
	932.6	Cu^0^	23.6% ± 0.7%	24.9% ± 4.3%
	933.8	CuO	39.4% ± 1.1%	32.8% ± 2.8%
	934.7	Cu(OH)_2_	35.4% ± 0.9%	41.5% ± 2.3%
Cu-DLC4	932.2	Cu_2_O	0.2% ± 0.1%	0.3% ± 0.1%
	932.6	Cu^0^	24.5% ± 4.0%	22.3% ± 0.9%
	933.8	CuO	28.0% ± 2.3%	27.0% ± 0.7%
	934.7	Cu(OH)_2_	49.3% ± 2.8%	50.5% ± 0.1%

## Data Availability

The data presented in this study are available on request from the corresponding author.

## References

[1] Organisation for Economic Co-operation and Development (OECD), European Commission (2016). Hip and Knee Replacement. Health at a Glance: Europe 2019: State of Health in the EU Cycle.

[2] (2018). Global Hip and Knee Replacement Market Set to Be Worth $20.4bn by 2028, Says GlobalData. https://www.globaldata.com/global-hip-knee-replacement-market-set-worth-20-4bn-2028-says-globaldata/.

[3] (2016). Annual Report 2016—American Joint Replacement Registry.

[4] Parisi T.J., Konopka J.F., Bedair H.S. (2017). What is the long-term economic societal effect of periprosthetic infections after THA? A Markov analysis. Clin. Orthop. Relat. Res..

[5] Brochin R.L., Phan K., Poeran J., Zubizarreta N., Galatz L.M., Moucha C.S. (2018). Trends in periprosthetic hip infection and associated costs: A population-based study assessing the impact of hospital factors using national data. J. Arthroplast..

[6] Romanò C.L., Scarponi S., Gallazzi E., Romanò D., Drago L. (2015). Antibacterial coating of implants in orthopaedics and trauma: A classification proposal in an evolving panorama. J. Orthop. Surg. Res..

[7] Romanò C.L., Tsuchiya H., Morelli I., Battaglia A.G., Drago L. (2019). Antibacterial coating of implants: Are we missing something?. Bone Jt. Res..

[8] Belanger M.C., Marois Y. (2001). Hemocompatibility, biocompatibility, inflammatory and in vivo studies of primary reference materials low-density polyethylene and polydimethylsiloxane: A review. J. Biomed. Mater. Res. A.

[9] Park B.S., Heo S.J., Kim C.S., Oh J.E., Kim J.M., Lee G., Park W.H., Chung C.P., Min B.M. (2005). Effects of adhesion molecules on the behavior of osteoblast-like cells and normal human fibro- blasts on different titanium surfaces. J. Biomed. Mater. Res. A.

[10] Gristina A.G. (1987). Biomaterial-centered infection: Microbial adhesion versus tissue integration. Science.

[11] Gristina A.G. (1994). Implant failure and the immuno-incompetent fibroinflammatory zone. Clin. Orthop. Relat. Res..

[12] Chu L., Yang Y., Yang S., Fan Q., Yu Z., Hu X.L., James T.D., He X.P., Tang T. (2018). Preferential Colonization of Osteoblasts Over Co-cultured Bacteria on a Bifunctional Biomaterial Surface. Front. Microbiol..

[13] Gotzmann G., Beckmann J., Wetzel C., Scholz B., Herrmann U., Neunzehn J. (2017). Electron-beam modification of DLC coatings for biomedical applications. Surf. Coat. Technol..

[14] Ren D.W., Zhao Q., Bendavid A. (2013). Anti-bacterial property of Si and F doped diamond-like carbon coatings. Surf. Coat. Technol..

[15] Ishihara M., Kosaka T., Nakamura T., Tsugawa K., Koga Y. (2006). Antibacterial activity of fluorine incorporated DLC films. Diam. Relat. Mater..

[16] Robertson S.N., Gibson D., MacKay W.G., Reid S., Birney R. (2017). Investigation of the antimicrobial properties of modified multilayer diamond-like carbon coatings on 316 stainless steel. Surf. Coat. Technol..

[17] Wu Y., Chen J., Li H., Ji L., Ye Y., Zhou H. (2013). Preparation and properties of Ag/DLC nanocomposite films fabricated by unbalanced magnetron sputtering. Appl. Surf. Sci..

[18] Hussain S., Pal A.K. (2007). Synthesis of composite films of mixed Ag–Cu nanocrystallites embedded in DLC matrix and associated surface plasmon properties. Appl. Surf. Sci..

[19] Su Y., Wang K., Gao J., Yang Y., Qin Y., Zheng Y., Zhu D. (2019). Enhance cytocompatibility and antibacterial property of zincphosphate coating on biodegradable zinc materials. Acta Biomater..

[20] Bociaga D., Komorowski P., Batory D., Szymanski W., Olejnik A., Jastrzebski K., Jakubowski W. (2015). Silver-doped nanocomposite carbon coatings (Ag-DLC) for biomedical applications Physiochemical and biological evaluation. Appl. Surf. Sci..

[21] Evrim B., Zeynep B., Ramazan E., Birgül Y.D. (2017). TiO_2_-NT electrodes modified with Ag and diamond like carbon (DLC) for hydrogen production by alkaline water electrolysis. Appl. Surf. Sci..

[22] Morrison M.L., Buchanan R.A., Liaw P.K., Berry C.J., Brigmon R.L., Riester L., Abernathy H., Jin C., Narayan R.J. (2006). Electrochemical and antimicrobial properties of diamondlike carbon-metal composite films. Diam. Relat. Mater..

[23] Constantinou M., Pervolaraki M., Nikolaou P., Prouskas C., Patsalas P., Kelires P., Giapintzakis J., Constantinides G. (2017). Microstructure and nanomechanical properties of pulsed excimer laser deposited DLC:Ag films: Enhanced nanotribological response. Surf. Coat. Technol..

[24] Baba K., Hatada R., Flege S., Ensinger W., Morimura T. (2013). Preparation and antibacterial properties of Ag-containing diamond-like carbon films prepared by a combination of magnetron sputtering and plasma source ion implantation. Vacuum.

[25] Świdwińska-Gajewska A.M., Czerczak S. (2014). Nanosilver harmful effect of biological activity. Med. Pr..

[26] Lansdown A.B.G., Anderson D., Waters M.D., Marrs T. (2010). Silver in Healthcare: Its Antimicrobial Efficacy and Safety in Use. Chapter 8—The Toxicology of Silver.

[27] Sengstock C., Braun D., Peetsch A., Diendorf J., Siebers B., Epple M., Köller M. (2012). The toxic effect of silver ions and silver nanoparticles towards bacteria and human cells occurs in the same concentration range. RSC Adv..

[28] Liu Y., Guo P., He X., Li L., Li H. (2016). Developing transparent copper-doped diamond-like carbon films for marine antifouling applications. Diam. Relat. Mater..

[29] Jonas J., Burns J., Abel E.W., Cresswell M.J., Strain J.J., Paterson C.R. (1993). Impaired mechanical strength of bone in experimental copper deficiency. Int. J. Nutr. Metab..

[30] Ewald A., Käppel C., Vorndran E., Moseke C., Gelinsky M., Gbureck U. (2012). The effect of Cu(II)-loaded brushite scaffolds on growth and activity ofosteoblastic cells. J. Biomed. Mater. Res..

[31] Barralet J., Gbureck U., Habibovic P., Vorndran E., Gerard C., Doillon C.J. (2009). Angiogenesis in calcium phosphate scaffolds by inorganic copper ion release. Tissue Eng. Part A.

[32] Thorwarth G., Saldamli B., Schwarz F., Jurgens P., Leiggener C., Haeberlen M., Assmann W., Stritzker B. (2007). Biocompatibility of doped diamond-like carbon coatings for medical implants. Plasma Process Polym..

[33] Bociaga D., Sobczyk-Guzenda A., Szymanski W., Jedrzejczak A., Jastrzebska A., Olejnik A., Swiatek L., Jastrzebski K. (2017). Diamond like carbon coatings doped by Si fabricated by a multi-target DC-RF magnetron sputtering method—Mechanical properties, chemical analysis and biological evaluation. Vacuum.

[34] Ferraria A., Carapeto A., Rego A. (2012). X-ray photoelectron spectroscopy: Silver salts revisited. Vacuum.

[35] Akhavan O., Ghaderi E. (2010). Self-accumulated Ag nanoparticles on mesoporous TiO_2_ thin film with high bactericidal activities. Surf. Coat. Technol..

[36] Castro C.A., Jurado A., Sissa D., Giraldo S.A. (2012). Performance of Ag-TiO_2_ photocatalysts towards the photocatalytic disinfection of water under interior-lighting and solar-simulated light irradiations. Int. J. Photoenergy.

[37] Biesinger M.C., Lau L.W.M., Gerson A.R., Smart R.S.T.C. (2010). Resolving surface chemical states in XPS analysis of first row transition metals, oxides and hydroxides: Sc, Ti, V, Cu and Zn. Appl. Surf. Sci..

[38] Ivanov-Omskii V.I., Panina L.K., Yastrebov S.G. (2000). Amorphous hydrogenated carbon doped with copper as antifungal protective coating. Carbon.

[39] Mazare A., Anghel A., Surdu-Bob C., Totea G., Ionita D. (2018). Silver doped diamond-like carbon antibacterial and corrosion resistance coatings on titanium. Thin Solid Films.

[40] Písařík P., Jelínek M., Remsa J., Mikšovský J., Šepitka J. (2017). Antibacterial, mechanical and surface properties of Ag-DLC films prepared by dual PLD for medical applications. Mater. Sci. Eng. C.

[41] Kwok S.C.H., Ha P.C.T., McKenzie D.R., Bilek M.M.M., Chu P.K. (2006). Biocompatibility of calcium and phosphorus doped diamond-like carbon thin films synthesized by plasma immersion ion implantation and deposition. Diam. Relat. Mater..

[42] Meškinis Š., Čiegis A., Vasiliauskas A., Šlapikas K., Tamulevičius S. (2015). Optical properties of diamond-like carbon films containing copper, grown by high power pulsed magnetron sputtering and direct current magnetron sputtering: Structure and composition effects. Thin Solid Films.

[43] Stoian A.B., Surdu-Bo C., Anghel A., Ionita D., Demetrescu I. (2019). Investigation of High Voltage Anodic Plasma (HVAP) Ag-DLC Coatings on Ti50Zr with Different Ag Amounts. Coatings.

[44] Rhee S.K. (2006). Critical surface energies of Al_2_O_3_ and graphite. J. Am. Ceram. Soc..

[45] Sun L., Guo P., Li X., Wang A. (2017). Comparative study on structure and wetting properties of diamond-like carbon films by W and Cu doping. Diam. Relat. Mater..

[46] Micali G., Grilli J., Marchi J., Osella M., Cosentino Lagomarsino M. (2018). Dissecting the Control Mechanisms for DNA Replication and Cell Division in *E. coli*. Cell Rep..

[47] McClintock M.K., Fahnhorst G.W., Hoye T.R., Zhang K. (2018). Engineering the production of dipicolinic acid in *E. coli*. Metab. Eng..

[48] Chan Y.H., Huang C.F., Ou K.L., Peng P.W. (2011). Mechanical properties and antibacterial activity of copper doped diamond-like carbon films. Surf. Coat. Technol..

[49] Cremet L., Broquet A., Brulin B., Jacqueline C., Dauvergne S., Brion R., Asehnoune K., Corvec S., Heymann D., Caroff N. (2015). Pathogenic potential of Escherichia coli clinical strains from ortopedic implant infections towards human osteoblastic cells. Pathog. Dis..

[50] Jung W.K., Koo H.C., Kim K.W., Shin S., Kim S.H., Park Y.H. (2008). Antibacterial activity and mechanism of action of the silver ion in Staphylococcus aureus and Escherichia coli. Appl. Environ. Microbiol..

[51] Yamanaka M., Hara K., Kudo J. (2005). Bactericidal actions of a silver ion solution on Escherichia coli, studied by energy-filtering transmission electron microscopy and proteomic analysis. Appl. Environ. Microbiol..

[52] Goudouri O.M., Kontonasaki E., Lohbauer U., Boccaccini A.R. (2014). Antibacterial properties of metal and metalloid ions in chronic periodontitis and peri-implantitis therapy. Acta Biomater..

[53] Hauser S., Jung F., Pietzsch J. (2017). Human endothelial cell models in biomaterial research. Trends Biotechnol..

[54] Rodríguez J.P., Ríos S., González M. (2002). Modulation of the proliferation and differentiation of human mesenchymal stem cells by copper. J. Cell. Biochem..

[55] Burghardt I., Lüthen F., Prinz C., Kreikemeyer B., Zietz C., Neumann H.G., Rychly J. (2015). A dual function of copper in designing regenerative implants. Biomaterials.

[56] Zhang X., Li J., Wang X., Wang Y., Hang R., Huang X., Tang B., Chub P.K. (2018). Effects of copper nanoparticles in porous TiO_2_ coatings on bacterial resistance and cytocompatibility of osteoblasts and endothelial cells. Mater. Sci. Eng. C.

[57] Hang R.Q., Gao A., Huang X.B., Wang X.G., Zhang X.Y., Qin L., Tang B. (2014). Antibacterial activity and cytocompatibility of Cu–Ti–O nanotubes. J. Biomed. Mater. Res. B.

[58] Lin Y., Xiao W., Bal B.S., Rahaman M.N. (2016). Effect of copper-doped silicate 13–93 bioactive glass scaffolds on the response of MC3T3-E1 cells in vitro and on bone regeneration and angiogenesis in rat calvarial defects in vivo. Mater. Sci. Eng. C.

[59] Huang Q., Liu X., Zhang R., Yang X., Lan C., Feng Q., Liu Y. (2019). The development of Cu-incorporated micro/nano-topographical bio-ceramic coatings for enhanced osteoblast response. Appl. Surf. Sci..

[60] G’erard C., Bordeleau L.J., Barralet J., Doillon C.J. (2010). The stimulation of angiogenesis and collagen deposition by copper. Biomaterials.

[61] Rafi A., Devaki R., Sabita K., Mohanty S., Rao P. (2013). Importance of serum copper and vascular endothelial growth factor vegf-a levels in postmenopausal bleeding. Indian J. Clin. Biochem..

[62] Li S., Xie H., Li S., Kang Y.J. (2012). Copper stimulates growth of human umbilical vein endothelial cells in a vascular endothelial growth factor-independent pathway. Exp. Biol. Med..

[63] Albers C.E., Hofstetter W., Siebenrock K.A., Landmann R., Klenke F.M. (2013). In vitro cytotoxicity of silver nanoparticles on osteoblasts and osteoclasts at antibacterial concentrations. Nanotoxicology.

[64] Marciano F.R., Bonetti L.F., Santos L.V., Da-Silva N.S., Corat E.J., Trava-Airoldi V.J. (2009). Antibacterial activity of DLC and Ag–DLC films produced by PECVD technique. Diam. Relat. Mater..

[65] Kalishwaralal K., Banumathi E., Pandian S.R.K., Deepak V., Muniyandi J., Eom S.H., Gurunathan S. (2009). Silver nanoparticles inhibit VEGF induced cell proliferation and migration in bovine retinal endothelial cells. Colloids Surf. B.

[66] Bostancıoğlu R.B., Peksen C., Genc H., Gürbüz M., Karel F.B., Koparal A.S., Dogan A., Kose N., Koparal A.T. (2015). Analyses of the modulatory effects of antibacterial silver doped calcium phosphate-based ceramic nano-powder on proliferation, survival, and angiogenic capacity of different mammalian cells in vitro. Biomed. Mater..

